# Artificial intelligence-based preoperative prediction system for diagnosis and prognosis in epithelial ovarian cancer: A multicenter study

**DOI:** 10.3389/fonc.2022.975703

**Published:** 2022-09-21

**Authors:** Meixuan Wu, Yaqian Zhao, Xuhui Dong, Yue Jin, Shanshan Cheng, Nan Zhang, Shilin Xu, Sijia Gu, Yongsong Wu, Jiani Yang, Liangqing Yao, Yu Wang

**Affiliations:** ^1^ Department of Obstetrics and Gynecology, Shanghai First Maternity and Infant Hospital, School of Medicine, Tongji University, Shanghai, China; ^2^ Department of Obstetrics and Gynecology, Renji Hospital, School of Medicine, Shanghai Jiaotong University, Shanghai, China; ^3^ Obstetrics and Gynecology Hospital, Fudan University, Shanghai, China

**Keywords:** artificial intelligence, epithelial ovarian cancer, blood biomarkers, diagnosis, prognosis, SHAP value

## Abstract

**Background:**

Ovarian cancer (OC) is the most lethal gynecological malignancy, with limited early screening methods and poor prognosis. Artificial intelligence technology has made a great breakthrough in cancer diagnosis.

**Purpose:**

We aim to develop a specific interpretable machine learning (ML) prediction model for the diagnosis and prognosis of epithelial ovarian cancer (EOC) based on a variety of biomarkers.

**Methods:**

A total of 521 patients with EOC and 144 patients with benign gynecological diseases were enrolled including derivation datasets and an external validation cohort. The predicted information was acquired by 9 supervised ML methods, through 34 parameters. Behind predicted reasons for the best ML were improved by using the SHapley Additive exPlanations (SHAP) algorithm. In addition, the prognosis of EOC was analyzed by unsupervised clustering and Kaplan–Meier (KM) survival analysis.

**Results:**

ML technology was superior to conventional logistic regression in predicting EOC diagnosis and XGBoost performed best in the external validation datasets. The AUC values of distinguishing EOC and benign disease patients, determining pathological type, grade and clinical stage were 0.958 (0.926-0.989), 0.792 (0.701-0.8834), 0.819 (0.687-0.950) and 0.68 (0.573-0.788) respectively. For negative CA-125 EOC patients, the AUC performance of XGBoost model was 0.835(0.763-0.907). We used unsupervised cluster analysis to identify EOC subgroups with significantly poor overall survival (p-value <0.0001) and recurrence-free survival (p-value <0.0001).

**Conclusions:**

Based on the preoperative characteristics, we proved that ML algorithm can provide an acceptable diagnosis and prognosis prediction model for EOC patients. Meanwhile, SHAP analysis can improve the interpretability of ML models and contribute to precision medicine.

## Introduction

Ovarian cancer (OC) ranks the fifth leading cause of cancer-related death in women (1). The American Cancer Society calculates that approximately 21,410 newly diagnosed OC cases and 13,770 deaths will happen in the United States in 2021 (1). According to the International Federation of Gynecology and Obstetrics (FIGO) staging criteria, epithelial ovarian cancer (EOC) can be divided into stages I, II, III and IV according to surgical pathological staging. With the increase of stage, the survival rate of patients significantly decreased (2). For EOC, preoperative diagnosis and regular follow-up after treatment mainly include the determination of serum biomarkers and imaging examinations. The serum biomarkers mainly include Carbohydrate antigen 125 (CA-125) and Human epididymis protein 4 (HE4), which have limited diagnosis values due to relatively low sensitivity (3). Traditional EOC therapies include either primary surgical complete cytoreduction followed by the combination platinum-taxane-based chemotherapy or neoadjuvant-based chemotherapy followed by the separate surgical cytoreduction and additional chemotherapy after surgery (4). Despite current advances in personalized treatments, EOC still holds a high recurrence rate after adequate treatments, because of its heterogeneous (5, 6). Moreover, due to the limitation of monitoring methods, many patients cannot be diagnosed in time, leading to poor survival (7). Therefore, the establishment and validation of the promising prognosis and risk stratification model are of great urgency to help clinical decision-making and improve survival in the realm of precision medicine for EOC.

Recently, with the innovation of electronic devices and the development of science and technology, the exploration and application of artificial intelligence (AI) in medicine have changed our understanding of the traditional medical world in the past years (8, 9). Machine learning (ML) is a branch derived from artificial intelligence, which builds data models to analyze, calculate and make predictions on huge clinical data. It presents its analysis results in the form of data or charts, to solve medical problems hidden behind the data (10). At present, ML model has been applied to disease screening and diagnosis. To a certain extent, the diagnostic efficiency and the prognosis of patients have been improved (11). For instance, Zhang L and colleagues developed a new algorithm to identify benign and malignant ovarian cysts by combining tumor markers with ultrasound images (12). Wang S et al. also created a new model to predict the recurrence of EOC by machine learning (13). In addition, AI is black-box prediction model, which cannot easily explain the reasoning process to clinicians. Therefore, effective interpretability can increase physicians’ trust in the models. A study assessed the quality of interpretability techniques and believed it is significant for the users of interpretable techniques to clearly state their interpretable focus (14). Recently, an explainable AI early warning score system for detection of acute critical illness has been proposed based on electronic health records, when maintaining high predictive performance (15). Monsarrat P et al. combined both ML and SHapley Additive exPlanations (SHAP) explainability algorithms to develop a new strategy for predicting periodontal health (16). Thus, we adopted ML predictive model and interpretable algorithm as a way to improve the reliability and clinical utility of the models. In this study, we are committed to using machine learning, mainly including Logistic regression (LR), Decision tree (DT), Random Forest (RF), Adaptive boosting (AdaBoost), Extreme gradient boosting (XGBoost), Gradient boosting machine (GBM), Naive Bayes (NB), Support Vector Machine (SVM), Elastic Net (EN) and Neural network (NNET) to explore the relationship among serum biological indicators and other clinical variables in EOC. Making a preoperative risk assessment is necessary to optimize patient management. However, we do not know the outcome of risk stratification for our dataset. Due to the unsupervised nature of the clustering, it can automatically reveal groups of biological significance for predicting overall survival (OS) and recurrence-free survival (RFS), regardless of the available knowledge that we have known. Therefore, we used unsupervised clustering to determine the groups as well as to identify features. In this study, we aimed to develop and validate machine learning prediction methods based on multiple blood biomarkers and clinical characteristics for estimation of diagnosis, clinical features (including pathological subtype, pathological grade, and clinical stage). And based on unsupervised machine learning, we hope to select personalized therapy by pretreatment prognosis stratification of EOC patients.

## Materials and methods

### Study population

We retrospectively screened the data of 443 patients confirmed EOC from Jan.2010 to Dec.2020 from our institution. Then, patients were excluded referring to the following criteria : (1) without incomplete clinical stage and histologic type (n=10), (2) with cancer coexistence or past medical history within 5 years(n=14). Finally, a total of 419 EOC patients were randomly matched with 113 benign gynecological diseases patients *via* age feature between Jun.2018 and Jun.2020 from our institution, approximately at the ratio of 4:1. Moreover, the external validation cohort that included 102 EOC patients and 31 benign gynecological diseases patients were enrolled from Obstetrics and Gynecology Hospital Affiliated to Fudan University from Jan, 2010, to Dec. 2020 to assess the performance of models. The analysis was approved by the Ethics Committee of Renji Hospital Affiliated to Shanghai Jiao Tong University School of Medicine, as well as the Ethics Committee of Obstetrics and Gynecology Hospital Affiliated to Fudan University.

### Feature selection and outcomes

The investigated dataset included 34 parameters: one clinical variable including age, 10 routine blood tests variables including White blood cell (WBC), Neutrophil (Neu), Lymphocyte (Lym), Monocyte (Mono), Eosinophil (Eo), Basophil (Baso), Red blood cell (RBC), Hemoglobin (Hb), Hematocrit (Hct), and Platelet (PLT), four tumor biomarkers variables including, Alpha-fetoprotein (AFP), Carcinoembryonic antigen (CEA), Carbohydrate antigen 19-9 (CA19-9), and Carbohydrate antigen 125 (CA-125), other blood features including Sodium (Na), Potassium (K), Chlorine (Cl), Urea nitrogen (UN), Creatinine (Cr), Uric acid (UA), glutamyl transpeptidase (GGT), Total protein (TP), Albumin (Alb), Alanine aminotransferase (ALT), Aspartate aminotransferase (AST), Alkaline phosphatase (ALP), Prealbumin (PA), globulin (GLOB), and Lactate dehydrogenase (LDH), Thrombin time (TT), Prothrombin time (PT), Fibrinogen (Fb) and Activated partial thromboplastin time (APTT). The mean imputation could be efficiently used for missing values of datasets. Meanwhile, this study analyzed the occurrence of 5 outcomes: disease diagnosis, histologic types and grade, clinical stage and prognosis of EOC patients.

### Model development and validation

In this study, the derivation cohort was randomly and repeatedly split into a training cohort (70%) which was used for developing the 9 ML models and tuning the parameter, and an internal validation cohort (30%) which was used for testing the models on unseen data to fine-tune the hyperparameters.

**Figure 1:**
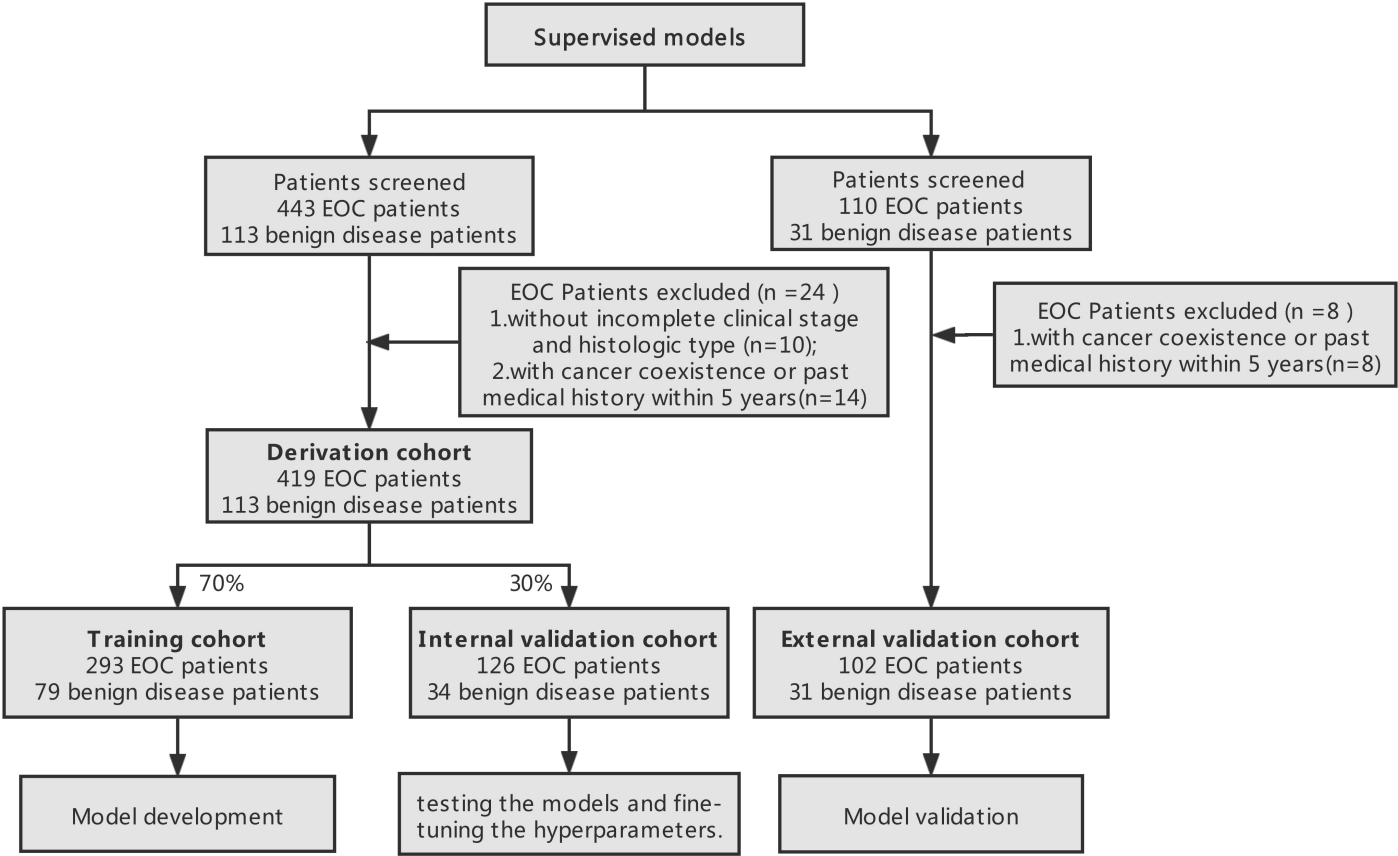
Study design process. Derivation datasets originated from the Department of Obstetrics and Gynecology, Renji Hospital and external validation cohort originated from Obstetrics and Gynecology Hospital Affiliated to Fudan University.

This resulted in the allocation of 293 patients with EOC and 79 people without EOC to the training cohort, and 126 patients with EOC and 34 people without EOC to the internal validation cohort. The study design process has been schematically shown in **Figure 1**.

### Supervised ML classifiers and unsupervised clustering

We applied nine types of supervised ML classifiers to model our cohorts: LR, DT, RF, GBM, XGBoost, AdaBoost, NB, SVM, and NNET. Classifiers were trained using k-fold cross-validation(k=5) to avoid overfitting and ensure the best hyper-parameter to evaluate the predictive result in the validation cohort. The final ML models were estimated by the confusion matrix metrics with the area under receiver operating characteristic (ROC) curve (AUC), accuracy, sensitivity, specificity and so on. In the performance comparison of ML algorithms, the closer the AUC is to 1, the better the classification model performs. All algorithms were implemented using R software (version 3.6.3) and the R package carets “e1071,” “rpart,” “randomForest,” “nnet,” “gbm,” “adabag,” “xgboost,” “Matrix,” “caret,” “tidyverse”. Multidimensional scaling (MDS) provides a set of datasets with the visible representation of the positional relationship. Subsequently, K-means unsupervised clustering algorithm was applied on the two scaling coordinates of MDS.

### XGBoost classifier and interpreting the model predictions

XGBoost algorithm, an integrated lifting algorithm, is implemented based on gradient tree boosting which has been proven to give many standard classification benchmarks with progressive achievements (14). The idea of ​​Boosting algorithm is to continuously improve and upgrade the weak classifiers, and integrate these classifiers to form a strong classifier. However, ML classifiers usually have distinctive black boxes and uninterpretable features, which means that the functions between the features and the responses are invisible to researchers (15–18). Here, SHapley Additive exPlanations (SHAP) method, evolved from cooperative game theory, was adopted to highlight the most contributing and important features, allowing the classifiers to generate global and individual interpretation of predicted outcome (19). SHAP analysis was implemented using R package “SHAPforxgboost” (https://CRAN.R-project.org/package=SHAPforxgboost).

### Statistical analyses

The Chi-square test and Wilcoxon test were calculated for categorical variables and non-normal continuous variables, respectively. Prognostic differences of Kaplan–Meier (KM) survival curves were compared through the Log-rank test. The EOC patients were categorized into three groups according to the optimal cutoff value for CA-125 and Alb, which were determined by the ROC curve. To evaluate the correlation between blood markers, Spearman rank coefficient was applied for the clustering important features. All analyses were two tailed and unpaired with the significance set at P < 0.05.

## Results

### Baseline characteristics

We summarized the clinical variable characteristics of 521 patients with EOC in **Table S1** and the outcome distribution of training and validation sets was shown in **Table 1**.

**Table 1:** Outcomes between training cohort and validation cohorts.

Characteristics	Training (n=372)	Internal validation (n=160)	External validation (n=133)
**Benign gynecological diseases** **EOC **	79 293	34 126	31 102
**Stage (%)**			
I	74 (25.3)	31 (24.6)	41 (40.2)
II	32 (10.9)	13 (10.3)	20 (19.6)
III	151 (51.5)	69 (54.8)	38 (37.3)
IV	36 (12.3)	13 (10.3)	3 (2.9)
**Histologic types (%)**			
Serous	207 (70.6)	97 (77.0)	63 (61.8)
Endometrioid	43 (14.7)	15 (11.9)	19 (18.6)
Mucinous	36 (12.3)	12 (9.5)	10 (9.8)
Clear	5 (1.7)	2 (1.6)	8 (7.8)
others	2 (0.7)	0 (0.0)	2 (2.0)
**Pathological grade (%)**			
G1	12 (4.1)	2 (1.6)	4 (3.9)
G2	28 (9.6)	19 (15.1)	2 (2.0)
G3	240 (81.9)	101 (80.2)	79 (77.5)
NA	13 (4.4)	4 (3.2)	17 (16.7)

### EOC diagnosis and ML models comparison

**Figure 2:**
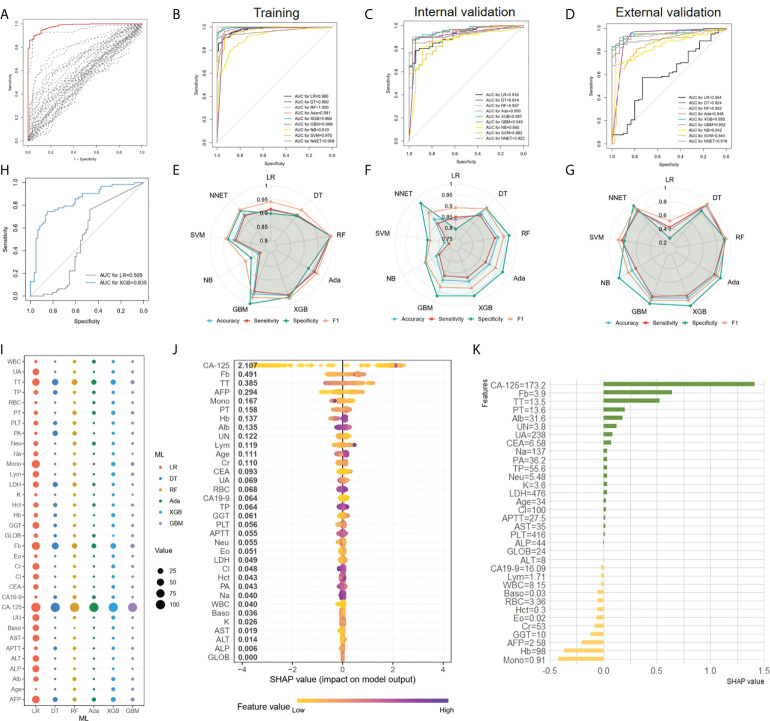
EOC diagnosis and ML models comparison **(A)**, ROC curves originated from multiple logistic regression for predicting EOC. The result of a multiple regression model using all 34 markers was indicated in the red line, whereas single regression results were represented by dashed lines. **(B–D)** Comparing the ROC curve of ML algorithms on the training set **(B)**, the internal validation set **(C)** and the external validation set **(D) (E–G)** The accuracy, sensitivity, specificity and F1 of each model depicted by radar plot in the training dataset **(E)**, the internal validation dataset **(F)** and the external validation dataset **(G)**. **(H)** Comparison the AUC of XGBoost and LR in the diagnosis of negative CA-125 EOC patients. **(I)**, The relative importance ranking of variables for predicting EOC and benign diseases responses was calculated with LR, DT, RF, XGBoost and GBM. **(J)**, The XGBoost model showed great impact in predicting outcomes. **(K)**, The individual prediction result with SHAP method.

In the first place, we compared multivariate logistic regression analysis based on age and 33 peripheral blood markers with univariate logistic regression analysis using each marker to investigate the utility of multivariate as a predictor of EOC features. **Figure 2A** illustrated the ROC curve originated from multiple logistic regression for predicting EOC based on multiple markers in all persons (red line), which was superior to those of any single regression depicted by dashed lines in **Figure 2A**. Thereafter, comparing the ROC curve of logistic regression and 8 ML algorithms on the training set (**Figure 2B**), we found ensemble learning methods including RandomForest, AdaBoost, XGBoost, and Gradient boosting machine exhibited better AUC values performance than other algorithms. In terms of prediction performance for EOC diagnosis, the AUC results of logistic regression and 8 ML algorithms on the internal validation set showed 0.957 (0.927-0.988) for XGBoost, 0.957 (0.928-0.986) for RF, 0.950(0.918-0.982) for AdaBoost, and 0.949(0.911-0.987) for GBM, 0.922 (0.882-0.962) for NNET, 0.916 (0.872-0.959) for LR, 0.914 (0.863-0.966) for DT, 0.882 (0.827-0.936) for SVM, and 0.860(0.7891-0.9317) for NB in **Figure 2C**. When applied to the external validation dataset, the AUC values were 0.958 (0.926-0.989) for XGBoost, 0.952 (0.918-0.987) for RF, 0.948(0.912-0.985) for AdaBoost, and 0.952(0.918-0.986) for GBM, 0.918(0.873-0.964) for NNET, 0.594(0.476-0.713) for LR, 0.924 (0.861-0.987) for DT, 0.844(0.794-0.894) for SVM, and 0.842 (0.771-0.914) for NB in **Figure 2D**, among which the first-rate prediction performance was observed with XGBoost. Furthermore, the accuracy, sensitivity, specificity and F1 of each model in the training dataset (**Figure 2E**), internal validation dataset (**Figure 2F**) and external validation dataset (**Figure 2G**) were depicted by radar plot, and the performances on RF, AdaBoost, XGBoost, and GBM were prominent and similarly. When considering the distribution of the EOC patients with negative CA-125(n=63)in the datasets, we used a stable and excellent XGBoost model and traditional LR model to predict the diagnosis of negative CA-125 EOC patients. We found that the performance of XGBoost model with the AUC of 0.835(0.763-0.907) was far superior to LR with the AUC of 0.505(0.398-0.612)(**Figure 2H**).

For the LR, DT, RF, Adaboost, XGBoost, and GBM models, we calculated the feature importance in the models using the built-in interpretation methods. Next, the normalized feature importance was used as the relative importance ranking of variables for predicting EOC and benign disease to compare the features in each model (**Figure 2I**), with CA-125, TT, and Fb as the main features in each model. At the same time, for the XGBoost model with the highest AUC values in the validation set, we used the SHAP value to interpret the model to observe features’ impact. Each point on the SHAP values figure represented a sample, and the color of the sample represented the value of the corresponding feature. In other words, if a sample was yellow, it denoted its feature value was low, and if purple, the feature value was high. All features and corresponding mean SHAP scores for the model sample were mapped to y-axis, and the SHAP values for each sample were mapped to x-axis. Apparently, the XGBoost model showed that CA-125, Fb, TT, AFP, were the characteristics of the XGBoost model with great impact in predicting outcomes (**Figure 2J**). For EOC diagnosis classification task, a positive SHAP value indicated EOC label with positive corresponding and a negative SHAP value indicated benign gynecological diseases. For instance, in **Figure 2J**, high CA-125 and Fb were positively correlated with predicting EOC. Subsequently, in order to investigate the characteristics contributions of the separate sample, we applied SHAP method to one case that was randomly chosen, and the result was shown in **Figure 2K**. Apparently, for this patient, CA-125 was positive with predicted result, as the value was 173.2U/ml, which was similarity with global interpretation. Meanwhile, mono (0.91 10^9/L) and Hb (98 g/L) were negative with the outcome. Thus, understanding the reasons behind the model’s individual prediction through SHAP method will improve clinicians’ satisfaction with the model, and trust model behavior and performance.

### Prediction of histologic types, grade and clinical stages of EOC with XGBoost classifier

**Figure 3:**
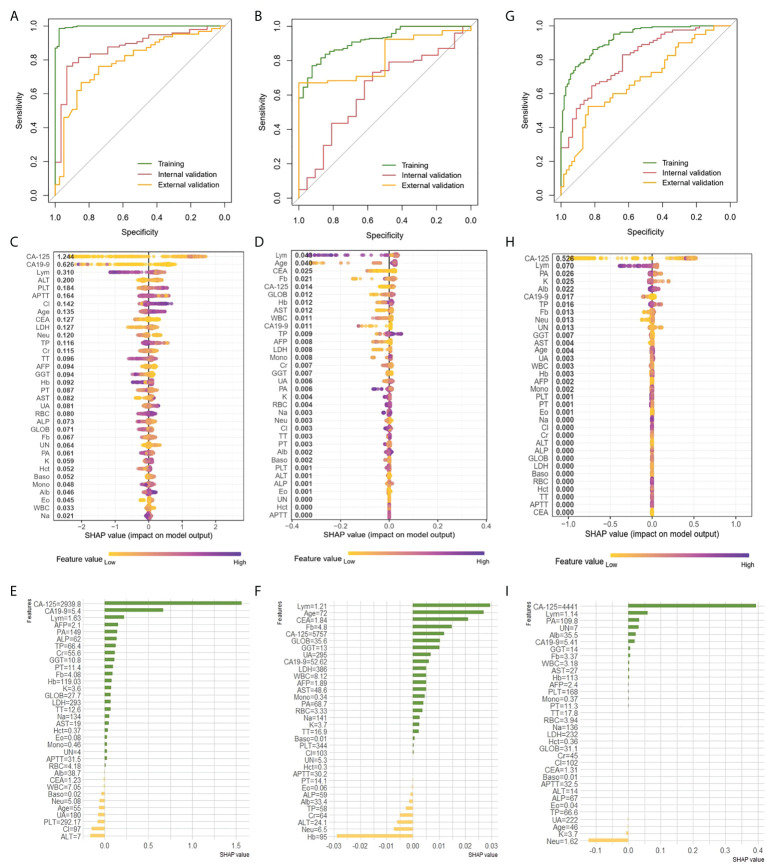
Prediction of histologic types, grade and clinical stage of EOC with XGBoost classifier A, B and G, ROC curve of the histologic types **(A)**, grade **(B)** and clinical stage **(G)** outcome in the training and validation cohorts. (**C, D,H)** Features ranking interpretation of the XGBoost model for predicting the histologic types **(C)**, grade **(D)** and clinical stage **(H)**. (**E, F, I**) Using SHAP to observe XGBoost model for predicting individual histologic types **(E)**, grade **(F)** and clinical stage **(I)**.

We attempted to preoperatively predict the histologic types of EOC disaggregated into serous and others, as well as grade disaggregated into G3 and others *via* using the age and 33 peripheral blood markers with the XGBoost classifier. The discriminative performance for the histologic type and grade outcome as expressed by the ROC curve in the training and two validation cohorts were shown in **Figures 3A, B**, respectively. AUCs of the XGBoost model for histologic types were 0.996 (0.992-1) in the training cohort, 0.872 (0.8-0.944) in the internal validation cohort and 0.792 (0.701-0.8834) in the external validation cohort. When the model was applied for histologic grade outcome prediction, AUC values were 0.916 (0.878-0.955) in the training cohort, 0.632(0.4986-0.765) in the internal validation cohort and 0.819 (0.687-0.950) in the external cohort. The accuracy, sensitivity, specificity and F1 were included in **Table 2**.

**Table 2:** Performance for XGBoost model.

	Accuracy (95% CI)	Sensitivity (95% CI)	Specificity (95% CI)	F1
**Histologic types**				
Training	0.983 (0.961-0.994)	0.986 (0.955-0.996)	0.977 (0.911-0.996)	0.987
Internal validation	0.802 (0.721-0.867)	0.763 (0.664-0.841)	0.931 (0.758-0.988)	0.856
External validation	0.735 (0.639, 0.818)	0.667 (0.536-0.777)	0.846 (0.688-0.936)	0.757
**Grade**				
Training	0.792 (0.740, 0.838)	0.771 (0.711-0.821)	0.923 (0.780-0.98)	0.865
Internal validation	0.705 (0.616, 0.784)	0.733 (0.634-0.814)	0.571 (0.344-0.774)	0.804
External validation	0.694 (0.585, 0.790)	0.671 (0.555-0.770)	1 (0.517-1)	0.803
**Clinical stage**				
Training	0.816 (0.767-0.858)	0.759 (0.690-0.817)	0.915 (0.841-0.958)	0.8402
Internal validation	0.762 (0.678-0.833)	0.829 (0.727-0.900)	0.636 (0.477-0.772)	0.8193
External validation	0.716 (0.618, 0.801)	0.525 (0.363-0.682)	0.839 (0.719-0.916)	0.5915

After running the XGBoost model for datasets, based on the SHAP algorithm, the features ranking interpretation of the XGBoost model for predicting the histologic types and grade were respectively shown in **Figures 3C, D**. We found CA-125, CA19-9 and Lym were the characteristics of the XGBoost model with the greatest impact in predicting histologic types outcomes (**Figure 3C**). The model for histologic types tended to associate high CA-125 with positive SHAP values, which meant positive correlation with outcomes. On the contrary, high CA19-9 and Lym were associated with negative correlation. For histologic grade, Lym, age, and CEA were the top three features of describing model (**Figure 3D**). Among them, high Lym was associated with negative correlation response, yet high age was associated with positive correlation. High Fb was negatively correlated with outcome, but it was also negatively correlated when it is low, even exceeding the high Fb. Next, we used individual SHAP value to observe XGBoost model for predicting histologic types and grade. For predicting histologic types, the patient with high CA-125 was correctly predicted as having high probability of serous (**Figure 3E**). And, ALT and Cl were mainly negative correlate with serous response for this patient. For predicting histologic grade of selected patient, the main characteristics were similar to global features. The main features were lym, age and CEA which were positively correlated with the outcome (**Figure 3F**).

As shown in **Figure 3G**, the AUCs of XGBoost classifier for distinguishing early-stage (stage I and II) from late-stage (stage III and IV) were performed 0.925 (0.8961-0.9538) for training cohort, 0.807 (0.729-0.884) for the internal validation cohort and 0.68 (0.573-0.788) in the external cohort. The accuracy, sensitivity, specificity, and F1 were included in the **Table 2**. Next, comparisons of the SHAP values performance of each feature were detailed in **Figure 3H**. Apparently, CA-125, Lym, PA and K were the four most important impact features of the XGBoost model in predicting clinical response. We could find there were general trends of features: higher CA-125 was associated with positive correlation, on the other hand, high Lym, PA, and K were associated with negative correlation of late stage. Same as before, we also estimated individual situation using SHAP method. For this case, the important characteristic was CA-125 (4441U/ml), which was positively correlated with the result (**Figure 3I**).

### Machine learning using unsupervised clustering analysis associated with prognosis

**Figure 4:**
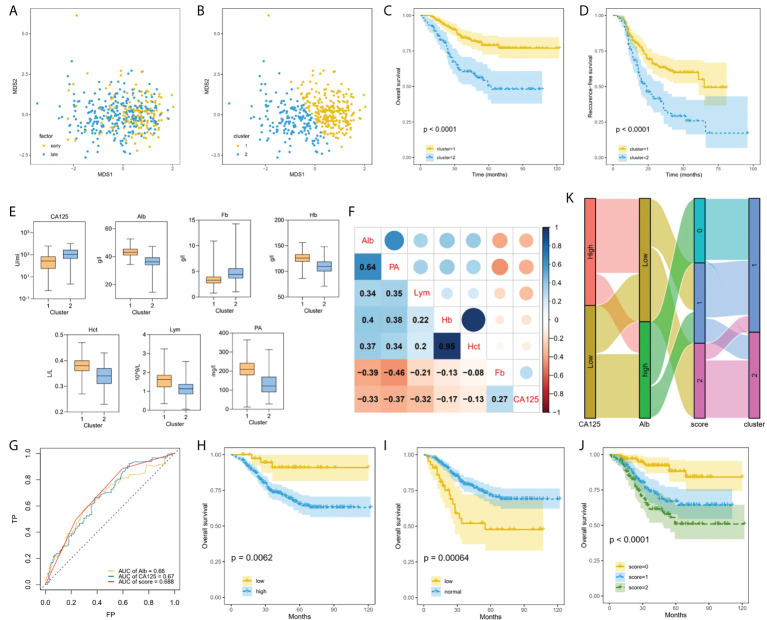
Machine learning using unsupervised clustering analysis associated with prognosis. **(A)**, Applied MDS technique to reduce dimension and generated MDS plot. **(B)**, EOC patients clustered into two groups using K-means method. **(C, D)** Kaplan–Meier curves showed OS **(C)** and RFS **(D)** of each cluster in all EOC. **(E)**, Box plots representing distribution of top seven differential blood markers between the cluster 1 and cluster 2. **(F)**, Correlation between top seven differential predictors evaluated using Spearman rank coefficient. **(G)**, Comparing the AUCs of CA-125, ALB and score. **(H–J)** Performing Kaplan–Meier method on the traditional CA-125 **(H)**, Alb **(I)**, and comprehensive score **(J)**. **(K)** Sankey plot showed the transition of the values of new CA-125, Alb, and score, and the proportions of clusters.

In the derivation datasets, 332 EOC patients had survival time follow-up information, of which 87 died, accounting for 26.2%. 301 cases knew whether there was recurrence information accurately, and 142 cases recurred, accounting for 47.2%. Data-driven groups were created using unsupervised machine learning. We initially applied MDS technique to reduce dimension to show a low-dimension (MDS1, MDS2) projection which could reserve as much as possible the distance among features in the original high-dimension datasets space, and generated MDS plot (**Figure 4A**). Then, K-means clustering analysis with K = 2 showed distinct clusters on the MDS data (**Figure 4B**). We found that most of the early-stage EOC were included in cluster 1, whereas late-stage EOC patients were widely distributed between clusters 1 and 2. Moreover, we also found a significant difference in OS (**Figure 4C**, p<0.0001) and RFS (**Figure 4D**, p<0.0001) between the clusters.

Multiple blood markers including CA-125, Lym, PA, Alb, Fb, Hb and Hct were significantly different in the two clusters (**Figure 4E**). To investigate the impact of these variables on prognosis, we initially performed Spearman correlation analysis, and we found there were strong positive correlations between Hb and Hct, and moderate positive correlations between PA and Alb (**Figure 4F**). Next, the AUC value of single significant variable in predicting 5-year survival was assessed by ROC analysis. We selected two variables CA-125 (AUC = 0.67) and ALB (AUC = 0.66) with AUC greater than 0.6 and without strong correlation. According to the ROC method, CA-125 = 510 U/mL and Alb=41.9 g/L were identified as the best cutoff value. We set CA-125 value greater than 510 U/ml as worth 1 score, for Alb, values greater than the cutoff point was considered 0 score, and vice versa, then, calculating their total scores. We compared the AUCs of CA-125, ALB and score, and found that the AUC value of comprehensive consideration of CA-125 and ALB was higher than that of single feature analysis (**Figure 4G**). We performed KM method on the traditional CA-125 with a normal value less than 35 U/ml (**Figure 4H**) and Alb with a normal value between 35 g/L and 55 g/L (**Figure 4I**), and the comprehensive score of CA-125 and Alb with 0, 1 and 2 points (**Figure 4J**). For EOC dataset, the comprehensive score achieved significantly different (p<0.0001). Sankey diagram directly shows the transition between the value including new CA-125, Alb and score and the two clusters (**Figure 4K**). As can be seen, 2 scores accounted for the highest proportion in cluster 2, which can help identify EOC patients at high risk of progressing to clusters with worse prognosis.

## Discussion

Compared with the relatively clear causes and screening methods of cervical cancer, screening and treatment of EOC need to be further researched. The biological indicators identified and processed by traditional medical statistical methods had limited for EOC screening. Meanwhile, AI has gradually been accepted by medical workers and used in decision-making assistance for some diseases (20–22). In gynecological tumors, the application of AI is also becoming increasingly prevalent (13, 23). Sanyal P et al. developed a deep learning model for interpreting cervical cell images to distinguish between benign and malignant cervical lesions with an accuracy of 94% (24). Laios A et al. used the clinical information of ovarian cancer patients as parameters to develop a model for predicting the negative resection margins of surgery through K- Nearest Neighbor, with an accuracy rate of 66% (25). Therefore, we can use machine learning to improve the accuracy of ovarian cancer prediction by existing screening methods and help manual decision-making, to reduce the occurrence of false-positive events and avoid unnecessary losses.

In this article, we have demonstrated the feasibility of using machine learning to develop a predictive model for EOC, using age and 33 peripheral blood parameters to analyze the diagnosis, clinical features (including pathological subtypes, pathological grade, and clinical stage) by supervised ML classifiers, as well as prognosis of patients *via* unsupervised clustering. In a previous study, AI system was used for diagnosis assessment of patients with EOC based on blood features through RF method (26, 27). In contrast, the best model, XGBoost, had a good AUC value in the internal and external validation cohorts when predicting EOC diagnosis in terms of our model establishment, which was better than LR and other 7 models including RF. The performance of external validation dataset proved the generalization ability of the models, which was not verified by the other machine learning models for ovarian cancer diagnosis before. In addition, compared with the conventional multiple logistic regression, XGBoost model also had an excellent diagnostic performance for negative CA-125 EOC patients, which can help identify false negative patients and avoid delaying treatment. Furthermore, we continued to analyze the pathology and clinical stage of EOC with the binary classification of XGBoost model. However, the performances of the validation sets were not as good as that of distinguishing benign from malignant. Therefore, we can attempt to use the deep learning to predict multi responses in the future.

However, the major issues in the use of ML in predicting response in the “black box” were complexity and opacity of algorithms, which limited their mainstream acceptance by the medical communities (28). Therefore, it is necessary to understand the clinical efficacies of the different models to generate clinical settings that help doctors make clinical decisions and develop optimal interpretation of ML model outcomes. Here, we utilized the model explanation algorithm, SHAP method, to determine the most important features for prediction. Research workers often use partial correlation diagrams or feature importance to explicate ML models before SHAP method was widely used. Through SHAP value, we can not only know the contribution of variables to prediction ability but also know the positive and negative correlation. As an instance, when analyzing EOC diagnosis, pathological type and clinical stage, high CA-125 was positively correlated with the prediction category, which was consistent with clinical cognition, and proved the availability of the model. Likewise, we also used the SHAP algorithm to explain the individual predicting reasons behind the model, which was difficult to achieve by traditional artificial evaluation. Different from the pure empiricism of traditional medical evaluation, we quantified the evaluation of individual patients, presented the evaluation results more intuitively, and promoted the development of precision medicine. It was the first time that we applied the SHAP algorithm to the XGBoost model of ovarian cancer, which enabled us to find potential indicators through the construction of data model, to help us understand the occurrence and development mechanism of ovarian cancer. It is hoped that more models and their interpretation algorithms will appear in the future, which can not only process high-throughput clinical data at the same time but also better improve the accuracy and interpretability of data prediction.

Moreover, recent studies have shown that the microenvironment of tumor growth plays an important role in the occurrence and development of tumor (29). Emerging research inspired us that we should not be limited to the detection of traditional tumor markers, such as CA-125, CA19-9, AFP and so on, but to explore the biological indicators related to the tumor growth microenvironment, which can also further judge the growth characteristics and biological behavior of tumors. For instance, in our previous work, we found that plasma fibrinogen to neutrophil ratio (F-NLR) can predict the prognosis of ovarian cancer to a certain extent (30). Machine learning can identify more biological indicators related to diagnosis and prognosis, to improve the accuracy and sensitivity of ovarian cancer screening. Consequently, our study used 9 ML models to predict the diagnosis of EOC with 34 features that collected were not merely limited to traditional tumor markers, but also included multiple biological indicators of peripheral blood, to achieve more accurate disease prediction. The machine learning methods in our study determined important factors for EOC diagnosis, such as FB, TT and Lym, in addition to the traditional CA-125.

As we mentioned previously, OC was commonly diagnosed in advanced stage, leading to a poor prognosis. Therefore, it is urgently needed for prognostic biomarkers that are noninvasive and reliable to help stratify patients. Clustering, as an unsupervised machine learning technique, has the ability to group observations based on similarities between measured features. Previously, unsupervised clustering was applied to multiple cancer types to identify clusters associated with prognosis and molecular subtypes (31–33). Unsupervised clustering analysis based on age and 33 preoperative blood markers was able to segregate EOC subgroups that were manifestly associated with prognosis, which could be recognizable preoperatively. In addition, we verified that comprehensive score of CA-125 and Alb was found useful in predicting disease overall survival outcome of EOC patients.

This study, however, also has some limitations. Firstly, the study was based on two-center databases, involving a relatively small number of patients. So, patients from more multiple sources are needed to verify the universal property of the model. Secondly, the retrospective nature of the study increased the possible risk for selection bias. In addition, although this study showed that machine learning can promote medical accurate decision-making to a certain extent, its clinical application and the responsibility of auxiliary medical decision-making still need to be further discussed.

In conclusion, we developed machine learning models to predict diagnosis and prognosis for EOC patients. ML can achieve more accurate preoperative evaluation, help doctors make decisions, avoid unnecessary surgery, guide the choice of different treatment schemes, and adapt to the development trend of contemporary precision medicine. We believe that future research can use AI by combining image data with serum biological indicators to develop new models and promote the diagnosis and treatment of ovarian cancer.

## Data availability statement

The datasets presented in this article are not readily available because the data of these findings cannot be shared at this time as the data also forms part of an ongoing study. Requests for data will be considered by the corresponding author after the publication of the study. Requests to access the datasets should be directed to Yu Wang, renjiwangyu@126.com.

## Ethics statement

Due to the retrospective nature of this study, participant informed consent was not required.

## Author contributions

MW and JY performed the study and wrote the manuscript. MW and JY interpreted the data and carried out statistical analysis. XD, SC, YZ, NZ, SX, SG, and YSW collected patients’ samples and clinical data. YW, JY, LY, and MW designed the study. MW drew the figures. All authors approved the final manuscript. Corresponding authors contributed equally to this work.

## Funding

This work was supported by the National Natural Science Foundation of China (Grant No. 82072866), Shanghai Special Program of Biomedical Science and Technology Support (Grant No. 21S31903600) and Clinical Scientific innovation and Cultivation Fund of Renji Hospital Affiliated School of Medicine, Shanghai Jiaotong University (Grant No. PYII20-02).

## Conflict of interest

The authors declare that the research was conducted in the absence of any commercial or financial relationships that could be construed as a potential conflict of interest.

## Publisher’s note

All claims expressed in this article are solely those of the authors and do not necessarily represent those of their affiliated organizations, or those of the publisher, the editors and the reviewers. Any product that may be evaluated in this article, or claim that may be made by its manufacturer, is not guaranteed or endorsed by the publisher.
