# Supplementary material for: Artificial intelligence-based preoperative prediction system for diagnosis and prognosis in epithelial ovarian cancer: A multicenter study
Source: Front Oncol. 2022 Sep 21;12:975703. doi: 10.3389/fonc.2022.975703 (PMC9532858; doi:10.3389/fonc.2022.975703)
Supplement: Supplementary file 1 [file Table_1.docx]

**Table S1: Baseline features of EOC patient cohorts**

| Characteristics | Training  (n=293) | Internal validation  （n=126） | External validation  (n=102) |
| --- | --- | --- | --- |
| Age (years) | 59.00 [52.00, 65.00] | 59.00 [51.25, 64.75] | 52.00 [46.00, 57.00] |
| Na (mmol/L) | 139.30 [137.10, 141.20] | 140.00 [138.00, 142.00] | 142.00 [142.00, 142.00] |
| K (mmol/L) | 3.67 [3.41, 3.90] | 3.67 [3.40, 3.86] | 4.00 [4.00, 4.00] |
| Cl (mmol/L) | 102.09 [100.00, 104.00] | 102.50 [100.00, 105.00] | 100.30 [100.30, 102.00] |
| UN (mmol/L) | 4.70 [3.70, 5.40] | 4.55 [3.70, 5.68] | 4.50 [3.90, 5.00] |
| Cr (umol/L) | 58.00 [51.00, 65.00] | 56.00 [49.85, 65.00] | 53.00 [49.00, 56.75] |
| UA (umol/L) | 277.15 [227.00, 322.00] | 263.50 [223.25, 313.00] | 268.00 [232.00, 286.50] |
| GGT (U/L) | 17.10 [13.00, 25.33] | 17.00 [12.77, 25.33] | 20.00 [14.00, 24.00] |
| TP (g/L) | 70.03 [66.50, 75.30] | 70.35 [65.12, 74.68] | 73.00 [70.00, 76.00] |
| Alb (g/L) | 40.90 [37.10, 44.40] | 41.50 [37.30, 44.50] | 43.00 [42.00, 45.00] |
| ALT (U/L) | 13.00 [9.00, 19.00] | 12.00 [10.00, 17.00] | 15.00 [11.25, 19.75] |
| AST (U/L) | 20.00 [15.00, 25.00] | 18.00 [15.03, 23.00] | 22.00 [18.00, 23.00] |
| ALP (U/L) | 73.00 [61.00, 86.00] | 72.00 [61.00, 91.50] | 80.00 [69.00, 90.50] |
| PA (mg/L) | 179.34 [130.90, 222.00] | 183.00 [136.30, 222.75] | 238.00 [213.00, 267.75] |
| GLOB (g/L) | 29.57 [26.10, 32.60] | 28.45 [25.72, 32.15] | 30.00 [27.25, 32.00] |
| LDH (U/L) | 223.00 [180.00, 279.00] | 214.00 [175.25, 264.25] | 208.00 [170.00, 242.25] |
| WBC (10^9/L) | 6.81 [5.67, 8.07] | 6.62 [5.58, 7.87] | 6.34 [5.21, 7.21] |
| Neu (10^9/L) | 4.91 [3.87, 5.93] | 4.56 [3.61, 5.72] | 4.30 [3.17, 5.29] |
| Lym (10^9/L) | 1.37 [1.04, 1.74] | 1.36 [1.08, 1.63] | 1.46 [1.16, 1.72] |
| Mono (10^9/L) | 0.45 [0.35, 0.55] | 0.44 [0.33, 0.56] | 0.38 [0.28, 0.45] |
| Eo (10^9/L) | 0.06 [0.03, 0.11] | 0.06 [0.03, 0.11] | 0.05 [0.02, 0.09] |
| Baso (10^9/L) | 0.02 [0.01, 0.03] | 0.02 [0.01, 0.03] | 0.02 [0.01, 0.03] |
| RBC (10^12/L) | 4.17 [3.84, 4.42] | 4.12 [3.80, 4.37] | 4.28 [4.08, 4.54] |
| Hb (g/L) | 121.00 [110.00, 129.00] | 119.52 [110.00, 129.75] | 123.00 [117.25, 131.00] |
| Hct (L/L) | 0.37 [0.34, 0.39] | 0.37 [0.34, 0.39] | 37.35 [35.80, 39.70] |
| PLT (10^9/L) | 276.00 [225.00, 347.00] | 270.50 [223.75, 333.75] | 252.00 [209.25, 283.00] |
| TT (sec) | 16.30 [14.60, 17.70] | 16.70 [15.33, 17.67] | 18.50 [17.02, 19.80] |
| PT (sec) | 11.40 [10.70, 12.30] | 11.45 [10.70, 12.28] | 11.00 [11.00, 11.00] |
| Fb (g/L) | 3.77 [2.99, 4.57] | 3.50 [2.93, 4.37] | 2.95 [2.60, 3.48] |
| APTT (sec) | 27.40 [26.00, 29.80] | 27.95 [26.20, 30.67] | 26.80 [25.30, 28.28] |
| AFP (ng/mL) | 2.60 [1.90, 3.59] | 2.49 [1.90, 3.30] | 3.34 [2.32, 19.26] |
| CEA (ng/mL) | 1.51 [0.82, 2.87] | 1.37 [0.87, 2.80] | 2.05 [1.30, 4.25] |
| CA19-9 (U/mL) | 12.80 [6.66, 47.20] | 11.70 [6.28, 37.45] | 22.04 [6.74, 149.60] |
| CA-125(U/mL) | 461.10 [117.20, 1,290.00] | 459.65 [122.08, 1,329.28] | 248.00 [59.29, 428.20] |

Abbreviations: EOC, Epithelial ovarian cancer; Na, Sodium; K, Potassium; Cl, Chlorine; UN, Urea nitrogen; Cr, Creatinine; UA, Uric acid; GGT, glutamyl transpeptidase; TP, Total protein; Alb, Albumin; ALT, Alanine aminotransferase; AST, Aspartate aminotransferase; ALP, Alkaline phosphatase; PA, Prealbumin; GLOB, globulin; LDH, Lactate dehydrogenase; WBC, White blood cell; Neu, Neutrophil; Lym, Lymphocyte; Mono, Monocyte; Eo, Eosinophil; Baso, Basophil; RBC, Red blood cell; Hb, Hemoglobin; Hct, Hematocrit; PLT, Platelet; TT, thrombin time; PT, Prothrombin time; Fb, fibrinogen; APTT, Activated partial thromboplastin time; AFP, Alpha-fetoprotein; CEA, Carcinoembryonic antigen; CA19-9, Carbohydrate antigen 19-9; CA-125, Carbohydrate antigen 125.
